# Ethical aspects of the abuse of pharmaceutical enhancements by healthy people in the context of improving cognitive functions

**DOI:** 10.1186/s13010-019-0076-5

**Published:** 2019-04-25

**Authors:** Tina Tomažič, Anita Kovačič Čelofiga

**Affiliations:** 1 Faculty of Electrical Engineering and Computer Science, University of Maribor, Institute for Media Communications, Koroška cesta 46, 2000 Maribor, Slovenia; 2 Alma Mater Europaea, Slovenska ulica 17, 2000 Maribor, Slovenia

**Keywords:** Cognitive enhancement, Nootropics, Ethics, Humanities, Society

## Abstract

Better memory, greater motivation and concentration lead to greater productivity, efficiency and performance, all of which are features that are highly valued in a modern society focused on productivity. In the effort for better cognitive abilities, otherwise healthy individuals use cognitive enhancers (also known as nootropics), medicines for the treatment of cognitive deficits of patients with various disorders and health problems, such as Alzheimer’s disease, schizophrenia, stroke, Attention Deficit Hyperactivity Disorder or ageing. The use of these is more common in professions with emphasised cognitive abilities, or in occupations that require more attention, focus and alertness. Their use is also associated with the general working population, in that they are supposed to use them to alleviate the effects of sleep deprivation and to cope with increasing workloads.

In the paper, we are addressing the ethical issue and the dilemmas of the use of pharmaceutical enhancements by healthy people who have no medical reason for taking such substances, in the context of improving their cognitive functions.

## Introduction

As a discipline, Neuroethics addresses a range of questions and issues generated by basic neuroscientific research, [1] exploring the application and implications of engaging Beuroscience in societal contexts [2], such as the use of Pharmaceutical Cognitive Enhancers (PCEs) by healthy people. PCEs, also known as nootropics^1^ or smart drugs, are basically regulated medicines. On the one hand, they are used as regulated prescription medicines that improve or reduce the risk of disease in people with a certain impairment or disease, and alleviate the existing or decreasing state of cognitive abilities [3]. PCEs are prescribed for the treatment of several disorders, including narcolepsy, sleep disorder, Attention Deficit Hyperactivity Disorder (ADHD) [4], Alzheimer’s disease, schizophrenia, etc. On the other hand, in the case of healthy people with no specific disorders or diseases, they can have the effect of strengthening or improving the state of their cognitive abilities [3], such as improving memory, attention, concentration, raising of alertness and intelligence. For example, “smart drugs” will be used to promote learning and clarify thinking, “happy pills” to increase mood and improve temperament, and “pep pills” to increase energy and maximise motivation [5].

Among the cognitive enhancers are also soft stimuli (such as coffee, energy drinks, sugar, food supplements, herbal preparations), nicotine, alcohol and illegal soft and hard drugs (ie marijuana, amphetamines, Lysergic acid diethylamide- LSD, heroin, cocaine, 3-Methylmethcathinone- 3MMC, psychedelic mushrooms, synthetic cannabinoids and Dimethyltryptamine -DMT).

Caffeine and nicotine are available without prescription, and are widely accepted as legitimate ways to help us be more focused, productive and awake. To a certain extent, cognitive abilities can be maintained, or even improved, primarily by means of an appropriate physical activity, various forms of meditation, yoga, sufficient amounts of rest and sleep, and quality nutrition. So, how to define a cognitive enhancer? It seems logical to point out that there are significant differences between natural enhancers and synthetic ones [6]. Now, there are pills to help you focus better and allow your mind to improve productivity and quality of work. It seems ideal, but there is a lot of disagreement about whether the use of these cognitive enhancers is fair, and whether such cognitive improvement is ethically acceptable [7].

For several reasons, unconventional forms of cognitive improvement deserve serious consideration: 1. There is not a great deal of “accumulated wisdom” about their potential use, safety, effectiveness or social consequences; 2. Potentially they can have a huge impact (consider the cost-benefit ratio for a low-cost pill that improves cognitive safety in comparison with years of additional education); 3. They are ethically disputable; 4. They face specific regulatory problems that may hinder their progress, and 5. Over time, they can have important consequences for society, and even, in the long run, for the future of mankind [8].

It is therefore necessary for PCEs to establish ethical, legal and medical rules and boundaries. Therefore, In the paper, we address the questions of the ethics of their use by healthy people, and what the consequences are for their enjoyment by society and our future in general.

## Humanistic aspect and prevalence of PCE abuse

In a product-oriented society, PCE abuse represents better memory, greater motivation and concentration, which are important advantages. In modern work environments, where employees are expected, and increasingly required, to work more efficiently and longer, and to be increasingly flexible and productive and more motivated, the desire for success and competitiveness is high [9]. The use of prescription drugs to improve cognitive effects is particularly common among individuals in cognitively demanding environments [10] related to intense intellectual work, high responsibility and stress, and long working hours. Surgeons, nurses, scientists, academics, pilots, flight controllers, firefighters, soldiers and drivers are good examples. Comprehensive studies of their actual prevalence among individual occupational groups have not yet been carried out. Most studies focus only on the use of medicines to improve cognitive function among students, but it is assumed that PCEs are more common in professions with emphasised cognitive abilities, or in occupations requiring more attention, focus and alertness. Often, these are professions where night work, shift or extended work is present. Finally, their use is also associated with the general working population, in that they are supposed to use them to alleviate the effects of sleep deprivation and to cope with increasing workloads [9].

Due to the lack of empirical research, motivations for the use of PCE among employees can only be guessed, based on broader social and labour trends and research among student populations. On the one hand, it undoubtedly promotes competitiveness and the desire for success of the individual, and on the other, it depends on the socio-economic factors [9] and the conditions and requirements of individual occupational groups.

Today, specific social circumstances are noticeable, for example the disintegration of traditional moral values, globalization on all levels of social life and consumer oriented society [11]. We can assume that capitalism is responsible for this situation in society to some extent. In any case, cognitive enhancement is about improving productivity, not about the consumption side of economic life. In any economy, people would want improved human productivity. That is why education is valued in every type of economic system, not just capitalism. In a society in which the Pharmaceutical industry offers medicines for every problem, a drug for increased productivity does not seem unusual [9]. This could encourage greater opportunities for individuals to have better education and profitable jobs. It could benefit society as a whole by creating a more informed population with a higher standard of living [12]. Last, but not least, the society promotes and demands (greater) efficiency, productivity and speed [9], which, of course, is also the desire of every individual who strives to improve their individual position. Namely, we need to perform larger quantities of work or more demanding tasks in the shortest possible time. We shorten our rest, the division between work and leisure is blurred, and there is growing competition among employees.

We live in a society where we need to be bigger, better and faster, and to be the best that we can be [13]. This is a society that glorifies competition and monetary success, and a decline in the use of tools that can bring competitive advantages can be considered a kind of moral failure [14]. It is a society where the philosophical concepts of the relation of brain to mind, and the values of ideas of Neuroscience and Neurotechnology can reflect in scientific, medical and socio-cultural realms [15], and show new challenges and opportunities [16].

## Ethical dilemmas: risks in non-medical use of PCE vs. benefits for society

There is a diversity of views on ethical, medical, legal and social issues relating to the non-medical use of PCEs by healthy people for the improvement of cognitive functions. Their use has received a lot of attention in scientific literature, in particular, because of their possible effects on productivity, efficiency, cognitive work and special professions due to personal and social savings [17], but also because of concerns about negative impacts on health, individual well-being and existing social structures and mechanisms [18].

Some studies show that, with the pharmacological enhancement of individual cognitive abilities, some of the other cognitive abilities may beimpaired, which cannot be affected seriously by the overall capacity of an individual in a given situation. At the individual level, adverse effects may occur in combination with certain other medicines, like drug-prohibited medicines, high-dose caffeine, or individuals who are more sensitive to substances or have a predisposition to certain mental illnesses and disorders. This is particularly problematic, because most of the PCEs are consumed without medical supervision. Therefore, many experts in this field point out that, in the context of the use of PCEs, the possibility of widely available and known quality information on effects and safe use should be available, as well as alternative approaches for strengthening cognition and consultation with a health professional, [3]. This would not lead to disorderly care and lack of medical monitoring.

Health may be compromised to some extent by cognitive improvement efforts if prescription drugs are obtained illegally on the black market, from unregulated and unofficial sources, and consumed without medical advice [19].

Namely, the demand for the PCEs and the current law have, between them, created a situation where the black market can flourish. For existing prescription-free medicines, such as Ritalin and Modafinil, this means that, instead of buying from unregulated sources such as friends or the Internet, medicines could become available from a legal source. This means that someone who takes PCEs knows exactly what they are taking. It is very difficult to obtain from an unregulated web source what the possible side effects are. If your doctor prescribes a medicine, you may be monitored to observe unwanted side effects and efficacy. A system in which a doctor has “control” over the patient’s use of cognitive medicine for improvement is safer than one where there is no experienced and professional input. There is currently an environment in society where the use of certain PCEs in some groups of people is widespread. The reality of the situation needs to be accepted, and everything that is done has to be as safe and as regulated as possible. By administering these medicines on a prescription, this will remove the negative effects of the “black market” of prescription drugs while avoiding the potential of abuse, so that they become freely available. In addition, by including them in a formal health framework, an environment is emerging that encourages research, and hopes to produce better PCEs in the future [20]. Legitimate improvements would certainly encourage development and use, and, in the longer term, would lead to cheaper and safer improvements, which means that it is necessary to regulate the field of PCEs in a legal way [8]. Thus, access to PCEs could be made through the health system, of course, only insofar as it could be justified scientifically that PCEs are effective and safe. On the other hand, Vince Cakic claims that to prohibit their use would mean going against individual freedom and reduce the benefits that they could bring to both the individual and society [13].

PCEs` supporters say that a clear link between the level of cognitive capacity and education, health, income, the range of available professions and social opportunities, as well as the vulnerability to various negative socio-economic outcomes, suggests that the use of PCEs could have positive impacts on an individual’s life. Enhanced cognitive abilities should not only mean greater competitiveness and an advantage in the labour market or in the social life of an individual, but also benefits at the level of society. On the one hand, they would be reflected in the social savings resulting from the reduced number of accidents and mistakes at work and in everyday life, due to improved alertness and attention, reduced costs and losses due to better memory. On the other hand, they could bring added value with a general increase in social productivity, and also possibly innovation and creativity. In this context, it is, of course, assumed that the PCEs are effective, safe, and only have mild side effects [3]. PCEs can also enable a company to compete more effectively, as companies are becoming significantly dependent on the consistent and sustainable mental performance of their employees in creating intellectual capital and competitive advantages. Toni Pustovrh claims that, until now, the army is one of the few professional organisations that has Regulations regarding the use of PCEs in specific combat situations, for example for long-range combat pilots [9].

It is noted that children in certain school districts are taking ADHD drugs for cognitive enhancement due to ambitious parents [21], which is a very sad fact and causes a lot of controversy in ethical and moral issues / dilemmas. Others feel that these medicines are often used to hide failures in the education system, by making non-achieving children more peaceful, rather than trying to develop learning methods that can accommodate a wider range of individual learning needs. However, if modern society requires much more study and intellectual concentration than was typical in an evolutionary adaptation environment, then it is not surprising that many people today are struggling to meet the demands of a school or workplace with great effort [8].

At universities today, it is no longer unusual for students to take ritalin while preparing for exams (not to mention caffeine, snacks with glucose and energy drinks). Should students be encouraged to take the means to improve the quality of their work (assuming they are safe and effective enough), for the same reason that we encourage them to make notes and begin to repeat the material early [8]? Many of the students taking PCEs have disclosed this information openly to their parents. Some parents have facilitated or encouraged them to continue using them, regardless of whether the student believes that they will help them manage their academic workload [22]. Students should know that prescription stimulants are not an easy way to get better grades. This is important, as research calls into question the ability of prescription stimulants to increase cognitive functioning in healthy people [23]. Some parents, however, investing financially in the success of their child, think differently, which is a very controversial ethical dilemma, but, at present, there are no formal rules forbidding their use in an academic setting [24].

Additionally, PCEs’ benefits, costs and risks are to be compared to the benefits, costs and risks of more “traditional” and established strategies and practices of cognitive enhancement, such as healthy eating, physical activities, appropriate sleep, meditation techniques and memory strategies, as well as with other technological means, cognitive improvements such as “brain training” games and (external) computing devices and software [25].

## Discussion

The use of prescription drugs for non-medical purposes has driven some of the controversy over cognitive enhancement [26]. No one knows what the long-term cognitive, affective and biological effects of PCEs in healthy people will be. Therefore, the non-medical use of PCEs deserves, for many reasons, an in-depth discussion, as we speak of medical, ethical, social and legal issues. As a society, we need to consider which forms of cognitive enhancement (e.g. pharmacological, exercise, lifelong learning) are acceptable, and for which groups under what conditions and by what methods we would wish to improve and flourish [27].

When talking about the medical issues of PCE use for healthy people, it is necessary to determine the safety and efficacy of these. Chronic use of psychotropic drugs could lead to the transformation of synapses and changes in neuronal circuits, and it is not known whether this would be beneficial or not. Of course, this concern is not only unique to improving medicines, but also to therapeutic medicines. It is still a matter for a doctor to prescribe a medicine with potential adverse effects for the therapeutic treatment of a mental disorder. Also, a doctor may prescribe a medicine with potentially adverse effects to improve normal mental function. If there is no better understanding of all the risks of the use of cognitive improvement medicines, potential damage due to the long-term use of these medicines justifies limiting their use to the short-term in specific circumstances, and only if there is a valid reason for their use [12]. At low doses, stimulants cause an increase in alertness, attention, and increased confidence and strength. When an overdose starts, excitement, confusion and psychosis develop. At very high doses, stimulants produce significant toxic effects, including coma, blood circulation and ultimate death. Higher doses of stimulants may also be used, leading to cognitive impairment and addiction [4]. However, the long-term effects of these forms of psychopharmacology are not known [12], and there is very limited scientific evidence to support cognitive performance in healthy individuals [28].

However, at the same time, there are a few obstacles in the development and use of cognitive improvements. One of the obstacles is the current licensing system for active substances and medical treatment. This system was designed to deal with traditional medicine, which aims to prevent, diagnose, treat, or alleviate disease. There is no room for improvement medicine in this context. For example, a company that produces pharmaceuticals could have major difficulties in obtaining regulatory approval for an agent whose sole use would be to improve cognitive performance in a healthy population. To date, any pharmaceutical agent on the market that gives the potential effect of cognitive improvement, has been developed to treat a pathological condition (such as ADHD, narcolepsy, or Alzheimer’s disease). The effects of cognitive improvement of these agents in healthy subjects are detected randomly, as unintended effects [8]. Progress in this area could be accelerated if pharmaceutical companies would concentrate directly on the development of PCEs for use in healthy populations, instead of acting indirectly, by proving that these drugs are also suitable for the treatment of a recognised disease.

The medical framework as a treatment for the disease not only causes problems for pharmaceutical companies, but also for users (“patients”) whose access to improvements often depends on whether they are able to find a doctor without prejudice who can prescribe the substance [8]. This causes inequity in access. People with large social capital and good information get access, while others are excluded. Furthermore, social concern is focused mainly on the safety of the purchase of prescription pharmaceuticals online or elsewhere on the black market from unregulated production sources.

Public health measures for preventing the non-medical use of pharmaceutical products for cognitive enhancement are exposed to some important challenges. First, it is important that health professionals are more aware of the non-medical use of pharmaceuticals for cognitive enhancement, such as misuse of prescription drugs and its risks. Second, the burden of responsible management can fall on healthcare professionals and patients, but the reality is that health service providers have little or no control over what is done when patients leave their offices. Public health information campaigns trying to prevent prescription misuse could target enhancement uses of prescriptions more directly. Furthermore, it is also important to increase public education, but, in raising public awareness of the cognitive strengthening of public health, it faces possible conflicts of values. It is, therefore, the important engagement of stakeholders and the general public, which could enrich the discussion and provide broader perspectives on the non-medical use of PCEs. Lastly, we could call for a public–private partnership between the Government and the Pharmaceutical industry to establish the safety and efficacy of currently well-used PCEs in healthy people. However, public health measures aim to prevent practices that could harm human health, such as the use of an over-the-counter medicinal product. On the other hand, raising consciousness may inadvertently promote forms of cognitive strengthening of healthy individuals. Public health interventions will need to consider carefully how they can play a role in cognitive strengthening. However, governments, medical professionals and research should account for possible, low-risk PCE use, and focus on providing solutions for individuals with problematic use patterns and lack of resources or lack of motivation to implement desired changes in patterns of PCE use [29]. Consequently, it is definitely important to examine the debate on PCEs in the public domain, and to inform and educate the public and all interested stakeholders better [30, 31].

## Conclusion

In the non-medical use of PCEs we speak of medical, ethical, social and legal dilemmas, that interfere strongly with the lives of individuals and the entire society. Regarding a better understanding of the trend, it is important to recommend that further research is needed, both in terms of the actual PCE’s neurophysiological effects, and the prevalence and socio-cultural specifics of their use by different populations of individual national environments. George Savulich and colleagues suggest, therefore, the main challenges: To investigate awareness of the risks posed by PCEs; to learn to manage the risks in the field of Safety and Health and promoting a healthy culture; to regulate the legalization of PCEs, and to explore the issues of the ethics of healthy people taking PCEs [30].

The fact is that taking PCEs is already something common in certain professional structures and social groups. So, more stringent regulation would most likely only strengthen the growth of the black market, where it is possible to find substances of questionable composition, quality and purity. Such a disorderly area does not benefit anyone, and it harms those who purchase these substances via the Internet or through foreign providers, i.e. beyond regulated sales and without medical supervision. The establishment of a legal market would, thus, allow for the control of potential side-effects and the monitoring of actual efficiency, and would give pharmaceutical companies the opportunity to concentrate on the development of medicinal products in relation to the actual purpose of use, and, most importantly, users would receive safe substances with legal and controlled production. It is, therefore, necessary to regulate the scope, and identify safe and efficient use of PCE frameworks to answer the dilemmas regarding their use, safety and efficacy and the social consequences. At the same time, non-medical use of PCEs should be able to educate health professionals, as well as the general public, to be acquainted better with the advantages and disadvantages of using PCEs, and acquire knowledge of alternative approaches to strengthening cognition, ranging from the relevant sleep, rest and physical exercise, to various techniques such as mindless drills, meditations and memory strategies.

## Abbreviations

ADHD: Attention Deficit Hyperactivity Disorder
DMT: Dimethyltryptamine
LSD: Lysergic acid diethylamide
PCE: Pharmaceutical Cognitive Enhancers

## Footnotes

1 Nootropics is an umbrella term that refers to smart drugs, stimulants (prescription medications, but also caffeine and nicotine), supplements (nutraceuricals), and other substances, such as racetams [30].
